# MALDI Profiling of Human Lung Cancer Subtypes

**DOI:** 10.1371/journal.pone.0007731

**Published:** 2009-11-05

**Authors:** Angelo Gámez-Pozo, Iker Sánchez-Navarro, Manuel Nistal, Enrique Calvo, Rosario Madero, Esther Díaz, Emilio Camafeita, Javier de Castro, Juan Antonio López, Manuel González-Barón, Enrique Espinosa, Juan Ángel Fresno Vara

**Affiliations:** 1 Laboratory of Molecular Pathology and Oncology, Unidad de Investigación, Hospital Universitario La Paz, Madrid, Spain; 2 Centro Nacional de Investigaciones Cardiovasculares (CNIC), Unidad de Proteómica, Madrid, Spain; 3 Unidad de Bioestadística, Hospital Universitario La Paz, Madrid, Spain; Queen Elizabeth Hospital, Hong Kong

## Abstract

**Background:**

Proteomics is expected to play a key role in cancer biomarker discovery. Although it has become feasible to rapidly analyze proteins from crude cell extracts using mass spectrometry, complex sample composition hampers this type of measurement. Therefore, for effective proteome analysis, it becomes critical to enrich samples for the analytes of interest. Despite that one-third of the proteins in eukaryotic cells are thought to be phosphorylated at some point in their life cycle, only a low percentage of intracellular proteins is phosphorylated at a given time.

**Methodology/Principal Findings:**

In this work, we have applied chromatographic phosphopeptide enrichment techniques to reduce the complexity of human clinical samples. A novel method for high-throughput peptide profiling of human tumor samples, using Parallel IMAC and MALDI-TOF MS, is described. We have applied this methodology to analyze human normal and cancer lung samples in the search for new biomarkers. Using a highly reproducible spectral processing algorithm to produce peptide mass profiles with minimal variability across the samples, lineal discriminant-based and decision tree–based classification models were generated. These models can distinguish normal from tumor samples, as well as differentiate the various non–small cell lung cancer histological subtypes.

**Conclusions/Significance:**

A novel, optimized sample preparation method and a careful data acquisition strategy is described for high-throughput peptide profiling of small amounts of human normal lung and lung cancer samples. We show that the appropriate combination of peptide expression values is able to discriminate normal lung from non-small cell lung cancer samples and among different histological subtypes. Our study does emphasize the great potential of proteomics in the molecular characterization of cancer.

## Introduction

In Western countries, lung cancer represents the leading cause of cancer-related death [1]. The 5-year overall survival rate is 15% and has not improved over many decades. This is mainly because approximately two-thirds of lung cancers are discovered at advanced stages. Furthermore, even among early-stage patients who are treated primarily by surgery with curative intent, 30–55% will develop and die of metastasis recurrence [2].

Today, lung cancer is classified according to histological criteria. The four main subtypes are: small cell lung cancer (SCLC), squamous cell carcinoma (SC), adenocarcinoma (AC), and large cell carcinoma (LC). Clinically, the last three are considered as non-small cell lung cancer (NSCLC), which accounts for about the 85% of all lung cancers [3]. Precise diagnosis and classification of cancers are critical for the selection of appropriate therapies. The advent of effective targeted therapies for lung cancer, such as the epidermal growth factor receptor inhibitors erlotinib and gefitinib, and the prospect of developing additional targeted therapies, has emphasized the importance of accurate diagnosis [4].

Proteomics is expected to play a key role in cancer biomarker discovery. Although it has become feasible to rapidly analyze proteins from crude cell extracts using mass spectrometry, sample complexity complicates these studies [5], [6]. Therefore, for effective proteome analysis it is essential to enrich samples for the analytes of interest [7]. Despite the fact that one-third of the proteins in eukaryotic cells are thought to be phosphorylated at some point in their life cycle, only a low percentage of the intracellular proteins is phosphorylated at any given time [8], [9]. Thus, a purification or enrichment step that isolates phosphorylated species would reduce complexity and increase sensitivity [10].

MALDI profiling is one of the most promising techniques to reduce the gap between high-throughput proteomics and clinic [7], [11]. MALDI MS can be used as a high-throughput method with outstanding sensitivity [6], enabling studies compromising large series of patients, and has the potential to revolutionise the early diagnosis of many diseases [12]. This capacity has been exemplified by MALDI protein profiling on tumor samples, which permitted the identification of markers that could be correlated with histological assessment and patient outcomes through statistical analysis [13], [14]. In this work, we applied phosphopeptide enrichment techniques to small human clinical samples based on Immobilized Metal Affinity Chromatography (IMAC) to reduce sample complexity. To detect new biomarkers, we have defined a data analysis workflow applying lineal discriminant-based and decision tree-based classification methods to analyze peptide profiles from human normal and cancer lung samples by mass spectrometry.

## Methods

### Ethics statement

At the time of initial diagnosis, all patients had provided consent in the sense that their tumour samples could be used for investigational purposes. Institutional approval from our ethical committee was obtained for the conduct of the study (Comité Ético de Investigación Clínica, Hospital Universitario La Paz). Data were analyzed anonymously. Patients provided written consent so that their samples and clinical data could be used for investigational purposes.

### Sample selection

Frozen samples from patients diagnosed with lung cancer: (15 Adenocarcinoma (AC), 15 Squamous cell carcinoma (SC) and 14 large cell carcinoma (LC) samples) and 15 normal lung (NL) samples were retrieved from the Department of Pathology of Hospital Universitario La Paz (Madrid, Spain). The histopathological features of each sample were reviewed by an experienced lung pathologist to confirm diagnosis and tumor content. Eligible samples had to include at least 50% of tumor cells.

### Total protein extraction, solubilization, and digestion

Samples were cut in a Leica CM3050S cryostat, obtaining 10 sections of 10 microns thickness of each. Tissue was processed with TRIzol reagent (Invitrogen, Carsbald, CA, USA) following the manufacturer's instructions. Pellets were resuspended in guanidine hydrochloride 6 M and heated 10 minutes at 95°C with agitation. Subsequently, 950 µl of 50 mM ammonium bicarbonate (pH 7–9) per sample were added. Protein sample concentration was measured by MicroBCA Protein Assay Kit (Pierce-Thermo Scientific, Rockford, IL, USA). Trypsin MS Grade Gold (Promega, Madison, WI, USA) was added to each sample to a 1∶50 relation. Digestion was carried out overnight at 37°C. The digested sample was divided into two aliquots.

### Parallel IMAC (PIMAC)

IMAC-Fe(III) based was performed in one aliquot of digested protein with PHOS-Select Iron Affinity Gel (Sigma-Aldrich, St. Louis, MO, USA) following the manufacturer's instructions. IMAC-based Ga(III) was performed in the other aliquot of digested protein with Phosphopeptide Isolation Kit (Pierce-Thermo Scientific, Rockford, IL, USA) following the manufacturer's instructions. Samples were stored at −20°C until further analysis.

### Phosphopeptide analysis by mass spectrometry

Peptide mixtures were vacuum dried and dissolved in a solution containing acetonitrile (30%) and TFA (0.1%). After bath-sonication (3 min), the peptides were 1∶1 mixed with either α-Cyano-4-hydroxycinnamic acid (CHCA) or 2,5-dihydroxybenzoic acid (DHB) used as matrices. A volume of 0.5 µl was deposited on the MALDI plate and was kept at room temperature until dried. MALDI-MS spectra (two replicates) were measured on a Bruker Ultraflex TOF/TOF MALDI mass spectrometer (Bruker-Daltonics, Billerica, MA, USA) [15] in the positive ion reflector mode. For protein identification, the peptide ions of interest were subject to MALDI-MS/MS analysis in the TOF/TOF mode, and the corresponding MS/MS spectra were transferred through the MS BioTools program (Bruker-Daltonics, Billerica, MA, USA) as inputs to search the NCBInr database using MASCOT software (Matrix Science, London, UK) [16].

### Differential m/z peaks selection

ClinProTools (CPT) software 2.1 (Bruker-Daltonics, Billerica, MA, USA) was used to select differential m/z peaks among samples subtypes (NL, AC, SC and LC). Spectra were processed as follows:

1. Normalization of all spectra to their Total Ion Count,
2. Recalibration of spectra on each other using the most prominent m/z peaks,
3. Baseline subtraction and m/z peak detection.

Once standardized and adjusted, CPT selects mass ranges which were considered as m/z peaks, and calculates peak areas for each spectrum [17]. Spectra were divided into two sets (Set 1 and Set 2), which include a different spot measurement per sample. Each set was divided in four spectra groups depending on the combinations between MALDI matrix and IMAC metal (Mx-Mt) used to obtain them (DHB-Fe, DHB-Ga, CHCA-Fe and CHCA-Ga). Each of these spectra groups were subsequently divided into histological subgroups (NL, AC, SC and LC) and analyzed separately by CPT. CPT settings were S/N>3 and Savitzky-Golay smoothing (1 cycle, m/z range = 5) [18]. The combination of these lists gives a combined Mx-Mt m/z peak list. Then we included all spectra of one Mx-Mt combination in CPT to measure all m/z peaks in the correspondent combined Mx-Mt m/z peak list. Peaks with Kruskal-Wallis p-value>0.1 were discarded. Common m/z peaks between two sets were selected. Finally, Pearson test between area values of each m/z peak achieved in Set 1 and Set 2 for all samples were performed and m/z peaks with r<0.4 were excluded. Thus, we obtained four final Mx-Mt lists of m/z peaks: DHB-Fe, DHB-Ga, CHCA-Fe and CHCA-Ga lists. Selected m/z peaks were considered consistent peaks.

### Discriminant Analysis and model generation

Discriminant Analysis of each final Mx-Mt m/z peak lists was performed in SPSS 9.0. m/z peaks included in each discriminant model were included in a second Stepwise Discriminant Analysis, which allowed the creation of a global discrimination model, including m/z peaks from all the Mx-Mt combinations.

### Supervised hierarchical clustering

Briefly, a vector is assigned to each pseudo-item, and this vector is used to compute the distances between this pseudo-item and all remaining items or pseudo-items using the same similarity metric that was used to calculate the initial similarity matrix. Analyses were performed in BRB-ArrayTools v3.6.1 developed by Dr. Richard Simon and Amy Peng Lang.

### Decision-tree ensemble algorithm

With the aim of selecting peaks that could differentiate between histological subtypes of lung cancer samples, we built a multi-peak classifier using AdaBoost decision tree-based classifier ensemble [19], [20]. Three independent analyses were performed: AC vs. (SC+LC), LC vs. (AC+SC) and SC vs. (AC+LC) using the final DHB-Ga m/z peak list. Normalized m/z peak intensity values from set1 were used as training set. Normalized m/z peak intensity values from set2 were used as test set. 200 iterations were performed in all cases. The area under the ROC (Receiving Operating Characteristic) curve (AUC) equals the probability of correctly classifying one pair of samples, each one for a separate class, and is used as a measurement of classifier performance (20). Statistical analyses were performed in R version 2.4 with the Boost software package version 1.0-0 and SPSS 9.0.

### Statistical analyses for identified peaks

After protein identification by MS/MS, ANOVA (when possible) and Kruskall-Wallis analyses were performed to assess differences in the expression of such proteins in the different histological subtypes. Mann-Whitney's U was applied to study differences between two subgroups after Kruskall-Wallis analyses. Statistical analyses were performed in SPSS 9.0.

### Immunohistochemistry

Formalin-fixed, paraffin-embedded tissue blocks, representative of NSCLC diagnosis, were retrieved following routine histopathological assessment. Sections were processed using a Dako Autostainer universal staining system (Dako, Glostrup, Denmark). For this study, 3.5-µm sections were immunostained with monoclonal antibody CK8 (1∶100 dilution; Novacastra, Newcastle upon Tyne, UK). Two tissue slices from each sample were evaluated.

## Results

The primary aim of the present study was to test whether tryptic peptide profiles, obtained from human normal and tumor lung samples using PIMAC and MALDI-TOF MS techniques, could discriminate Normal Lung (NL) from lung cancer, as well as between the most common lung cancer histological subtypes: AdenoCarcinoma (AC), Large Cell carcinoma (LC) and Squamous Cell carcinoma (SC). Only 49 from 59 samples were selected for the following analysis because samples without a minimum content of 50% tumor cells were discarded. Thus, 15 NL, 14 AC, 9 LC and 11 SC samples were subsequently analyzed. The mass spectrum generated for each sample typically contained several hundreds of peaks with S/N>3 [5].

Mass signal intensities of tryptic peptides derived from complex protein mixtures are mediated by several factors, namely relative protein concentration, varying enzymatic digestion efficiency, and sequence-dependent desorption/ ionization efficiencies. We performed a highly reproducible spectra processing procedure to obtain peak profiles with a high degree of concordance in the sample series. Consistent m/z peaks were selected following these criteria: mass peaks had to be present in both sample spots and Pearson's correlation between intensities of each peak achieved in Set 1 and Set 2 for all samples had to be >0.4. Mean Pearson's correlation coefficient was 0.8 for DHB peaks and 0.65 for CHCA peaks. An additional requirement (Kruskal-Wallis p-value<0.1) was applied in order to include peaks with discriminatory power between the sample subtypes. These criteria provided a consistent and reproducible methodology, as shown by mean Pearson's correlation coefficient of selected mass peaks.

We have investigated the overlap between peaks selected by each of the Mx-Mt combinations (Figure S1). Overall, 97 consistent mass peaks were identified across the four Mx-Mt combinations. Regarding MALDI matrices, 81 peaks were measured in DHB and 41 in CHCA analyses. Contrastingly, 80 peaks were measured in Ga-based IMAC and 42 in Fe-based IMAC analyses. In both cases, 25 overlapping peaks were found. Only four peaks were consistently present across all the Mx-Mt combinations.

Once the consistent peaks had been selected, a Stepwise Discriminant Analysis was performed in each final Mx-Mt peak list. Therefore, four discriminant models were constructed and the mass signals involved in each model are listed in Table S1. All these discriminant models were able to classify the samples into four groups, corresponding to NL, AC, SC and LC. Percentages of correctly classified samples by each model and leave-one-out cross-validation percentages of correctly classified samples are displayed in Table S1. A second Stepwise Discriminant Analysis was performed with peaks included in the four Mx-Mt Discriminant models (22 peaks) to avoid including noisy mass signals in the analysis. The Global Model included 9 m/z peaks and correctly classified 98.0% of the samples (48 of 49) in the LOOCV.

**Figure 1:**
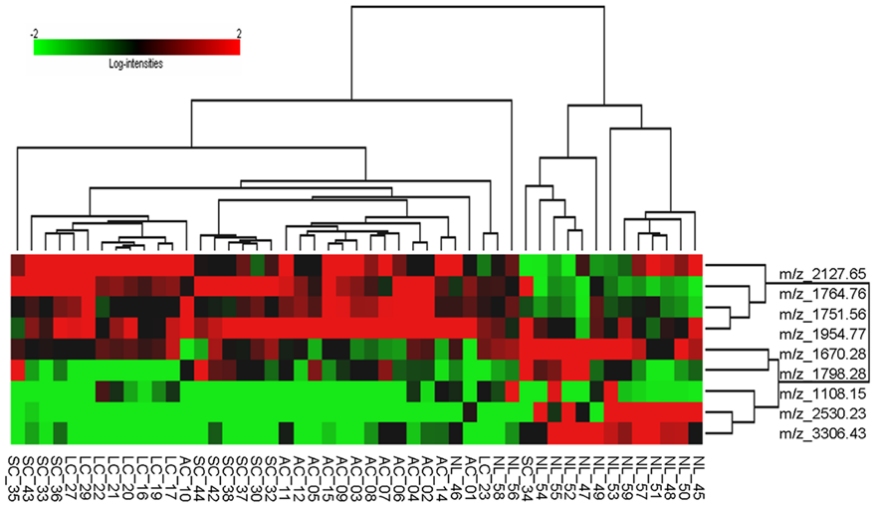
Hierarchical clustering analysis. Heat Map of the Supervised Hierarchical Centroid Linkage Clustering of normalized m/z peak areas, in two dimensions, for the 49 samples and the 9 m/z peaks included in the global discriminant model.

We performed a Supervised Hierarchical Centroid Linkage Clustering using the 9 peaks included in the Global Model. As shown in Figure 1, there are two main clusters, separating normal lung samples from most tumor samples. However, there is not perfect separation between histological subtypes. With the aim of selecting mass signals that could characterize samples from one histological subtype when compared with the other subtypes of NSCLC samples, AdaBoost decision tree-based classifier ensemble was performed. Three independent analyses were performed: AC vs. (SC+LC), LC vs. (AC+SC) and SC vs. (AC+LC), using data in Set 1 as training set and data in Set 2 as test set from the final DHB-Ga peak list. The area under the curve (AUC) from ROC was calculated for each comparison in both training and test set. The relative influence of each peak in model generation was obtained. The area under the ROC curve and top peaks for each comparison are shown in Table 1.

**Table 1:** Area under the ROC curve and top AdaBoost selected m/z peaks for each comparison.

Comparison	AUC training set	AUC test set	Top Peak list (m/z)
AC vs. (SC+LC)	0.982	0.961	2202.42, 1515.96, 1535.85, 2005.08, 2780.59
LC vs. (AC+SC)	0.991	0.871	1900.24, 2127.38, 2060.31, 2611.60, 1595.85
SC vs. (AC+LC)	1.000	0.893	2465.46, 2611.60, 2202.42, 2946.71, 2273.30

Adenocarcinoma (AC), Squamous cell Carcinoma (SC), Large cell Carcinoma (LC), Normal Lung (NL).

MS/MS identification of some m/z peaks selected by discriminant and AdaBoost analyses was performed by MALDI-TOF/TOF (Table S2). In order to evaluate differences in identified peptide signals among histological subtypes, ANOVA and Kruskal-Wallis analyses were performed. β-globin mass signals showed a significantly decreased intensity in tumor samples when compared with normal lung ones, while GAPDH and β-actin peaks showed increased intensity in tumor samples. CK8 peak intensity decreased in large cell carcinomas when compared with adenocarcinoma and squamous cell carcinoma samples.

**Figure 2:**
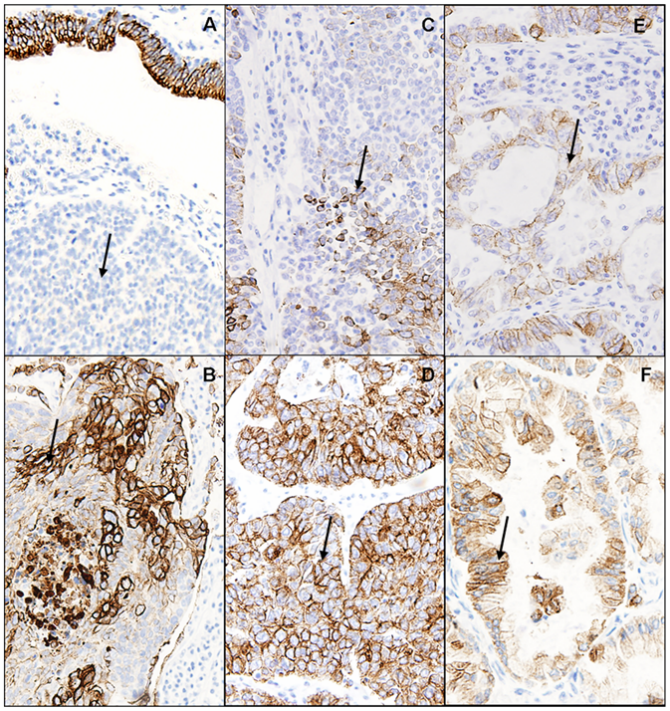
CK8 immunostaining. CK8 immunostaining (Magnification×40). Arrows point to tumoral cells. (A) Squamous cell carcinoma of the lung showing negative stained tumor cells. Lung epithelium shows positive staining. (B) Squamous cell carcinoma of the lung positively stained. (C,D) Large cell carcinoma of the lung showing different degrees of positive staining. (E,F) Adenocarcinoma of the lung showing different degrees of positive staining.

The pattern of expression by immunohistochemistry (IHC) of some of these markers was analyzed. The Human Protein Atlas (http://www.proteinatlas.org/) [21] shows expression and localization of proteins in a large variety of human normal and cancer tissues, as well as cell lines with the aid of IHC. IHC expression profiles for β-actin and GAPDH were evaluated on this useful database. There is an increased expression of β-actin in some lung cancer samples when compared with normal ones. However, GAPDH expression in lung cancer is highly variable. Additionally, we performed IHC analysis of CK8 expression in five AC, LC and SC samples. Positive cells for CK8 immunostaining were found in all LC and AC samples. By contrast, only three of five SC samples showed positive staining. Positively stained samples showed on average 20–70% stained cells (Figure 2).

## Discussion

Global gene-expression profiling has improved our understanding of the histological heterogeneity of non–small cell lung cancer and has identified potential biomarkers and gene signatures for classifying patients with significantly different survival outcomes [22]. A comprehensive understanding of the mechanisms behind carcinogenesis, tumor progression, and metastasis will require an in-depth analysis of not only the genome, but also the proteome [23]. Analyses at the gene level cannot detect the biologic subtleties introduced through post-translational modifications of proteins and thus requires a proteomic approach [5], [24].

Reproducibility has been shown to compromise protein profiling in all stages, from peptide isolation methods to sample spectra acquisition and processing [5], [11], [25], [26]. In this study, we have applied phosphopeptide enrichment chromatographic techniques to reduce the complexity of human lung cancer samples and analyzed isolated peptides by MALDI-TOF MS. We describe a mass peak selection method which yields a reproducible peptide profile from MALDI MS experiments using ClinProTools. Groseclose et al. described one limitation of using CPT is that peaks which may be significant among a small subset of spectra in a group, might become insignificant when averaged with the other spectra in that group [5]. In order to evaluate as many peaks as possible, we performed a previous step in the peak selection using CPT. In each Mx-Mt analysis, all spectra from a single sample subtype were introduced in CPT, obtaining a subtype characteristic peak list. Once all subtype lists were obtained, a new list was generated by combination, including all peaks present in these subtype lists. Afterwards, spectra from all sample subtypes were included in CPT, and all peaks in this combined list were measured. We confirmed that some discriminant peaks were excluded when spectra from all sample subtypes are included directly in CPT and standard analysis is performed.

It is noteworthy that when using DHB as a MALDI matrix provided a higher number of mass peaks as compared to CHCA. Likewise, the Ga-based IMAC approach produces more mass signals as compared to the Fe-based assay. In addition, the peak lists derived from DHB spectra showed a higher mean correlation between data sets. These results suggest that MALDI analyses using Ga-based IMAC and DHB as MALDI matrix are more reproducible and provide a higher number of mass signals. The peaks identified derived from highly expressed proteins and the remaining discriminating peptides could not be identified by MALDI MS. Alternative identification strategies should be tested in order to increase identification of low-intensity signals in MALDI MS studies.

Discriminant analyses allowed us to separate normal lung and NSCLC samples and to identify the peptides which best discriminated between normal and diseased tissues, as shown by clustering analysis (Figure 1). However, this task is not usually problematic due to the important differences between normal and cancer tissues. What proves trickier is finding differences between distinct histological subtypes. As showed in Figure 1, there are two main clusters of lung cancer samples, including adenocarcinomas and large cell carcinomas separately, but squamous cell carcinoma samples are splitted between these clusters.

It has been described that ensemble classifiers outperform single decision trees classifier by having greater accuracies and smaller prediction errors when applied to proteomics datasets [27]. So, we tested if AdaBoost analyses could classify the different NSCLC samples correctly. Our results suggest that AdaBoost can discriminate samples of one lung cancer histological subtype from the other two. The use of technical replicates as test set allowed us to assess the robustness of the methodology employed.

Our data suggest that both GAPDH and β-actin have a significantly increased expression in lung cancer samples. Overexpression of GAPDH in human lung cancers was described previously by Tokunaga et al [28] and there are many publications showing increased expression of GAPDH in breast [29], pancreatic [30] and cervical [31], [32] human cancers. On the other hand, several studies indicated that β-actin was differentially expressed in human cancer (reviewed in 28). Both proteins showed increased levels in rat hepatoma [33]. Moreover, IHC expression profiles for β-actin and GAPDH, assessed in the Human Protein Atlas, were highly variable in lung cancer samples. These results question the use of these proteins as housekeeping products in proteomic analyses of cancer samples.

Cytokeratin 8 (CK8) is a type II intermediate filament protein that is persistently expressed in most epithelial malignancies [34], including all NSCLC subtypes [35]. Increased levels of CK8 in sera have been associated with tumor progression and decreased survival in patients with NSCLC [36]. In contrast with these reports, we did not observe increased expression of CK8 in tumor samples by MALDI-MS analyses. However, we found out that CK8 levels are decreased in large cell carcinoma samples when compared with normal lung.

To assess the utility of CK8 expression as a biomarker of large cell carcinomas, we performed IHC analyses of CK8 expression in 15 lung cancer samples (five AC, five LC and five SC). In our opinion, no conclusion could be made about the relationship between IHC and peptide expression profiling from our data. This difference between techniques could be due to phosphopeptide enrichment prior to sample analysis or could imply that MS approaches are more sensitive than IHC. The peptide identified by MALDI MS/MS (DVDEAYMNKVELES) contains a potential phosphorylation site at Tyr204, related to phosphorylation by oncogenic kinases [37]. Previous studies assessing the utility of CK8 as a biomarker in lung cancer did not include any large cell carcinoma [35], [36].

The study has some constraints. Thus, there is limited capacity to identify minor mass peaks based on MS/MS analysis of relatively complex peptide mixtures. However, MALDI MS has some advantages for biomarker discovery: protein expression and relative quantification data can be generated for multiple patient tissue samples in a single experiment. On the other hand, comparison of IHC and peptide profiling expression values relationship should be done carefully, as it seems that prior affinity enrichment of samples could introduce some bias.

However, our study does emphasize the great potential of proteomics in the molecular characterization of cancer. Identification of differentially expressed proteins by PIMAC and MALDI-TOF/TOF MS was performed on fractionated tryptic digests derived from small amounts of tissues obtained from normal lung and NSCLC samples. Using an optimized sample preparation method and a careful data acquisition strategy, we overcame the major challenge of reproducibility of MALDI MS-based peptide profiling. Regardless of the nature of the peptides identified by MS/MS, the appropriate combination of peptide expression values is able to discriminate normal lung from NSCLC samples and among the different NSCLC histological subtypes. Future studies are aimed at establishing peptide profiling as a useful tool in the discovery of novel biomarkers with potential diagnostic or theragnostic relevance.

## Supporting Information

Figure S1 Venn diagrams showing m/z peaks overlapping between final m/z peak lists from: (A) four different Mx-Mt combinations, (B) IMAC resins, and (C) MALDI matrices.
(0.04 MB PPT)

Table S1 Percentages of correctly classified samples, leave-one out cross-validation percentages of correctly classified samples and m/z peaks included in each Mx-Mt combination discriminant model. Peaks in bold are also included in the 9 m/z peaks global discrimination model.
(0.03 MB DOC)

Table S2 Differentially expressed peptide masses from the CHCA-MALDI spectra identified by MALDI-TOF/TOF and MASCOT search engine. Individual MASCOT ions scores are significant (p<0.05).
(0.03 MB DOC)
