# Supplementary figures and images for: MALDI Profiling of Human Lung Cancer Subtypes
Source: PLoS One. 2009 Nov 5;4(11):e7731. doi: 10.1371/journal.pone.0007731 (PMC2767501; doi:10.1371/journal.pone.0007731)

## Slide 1
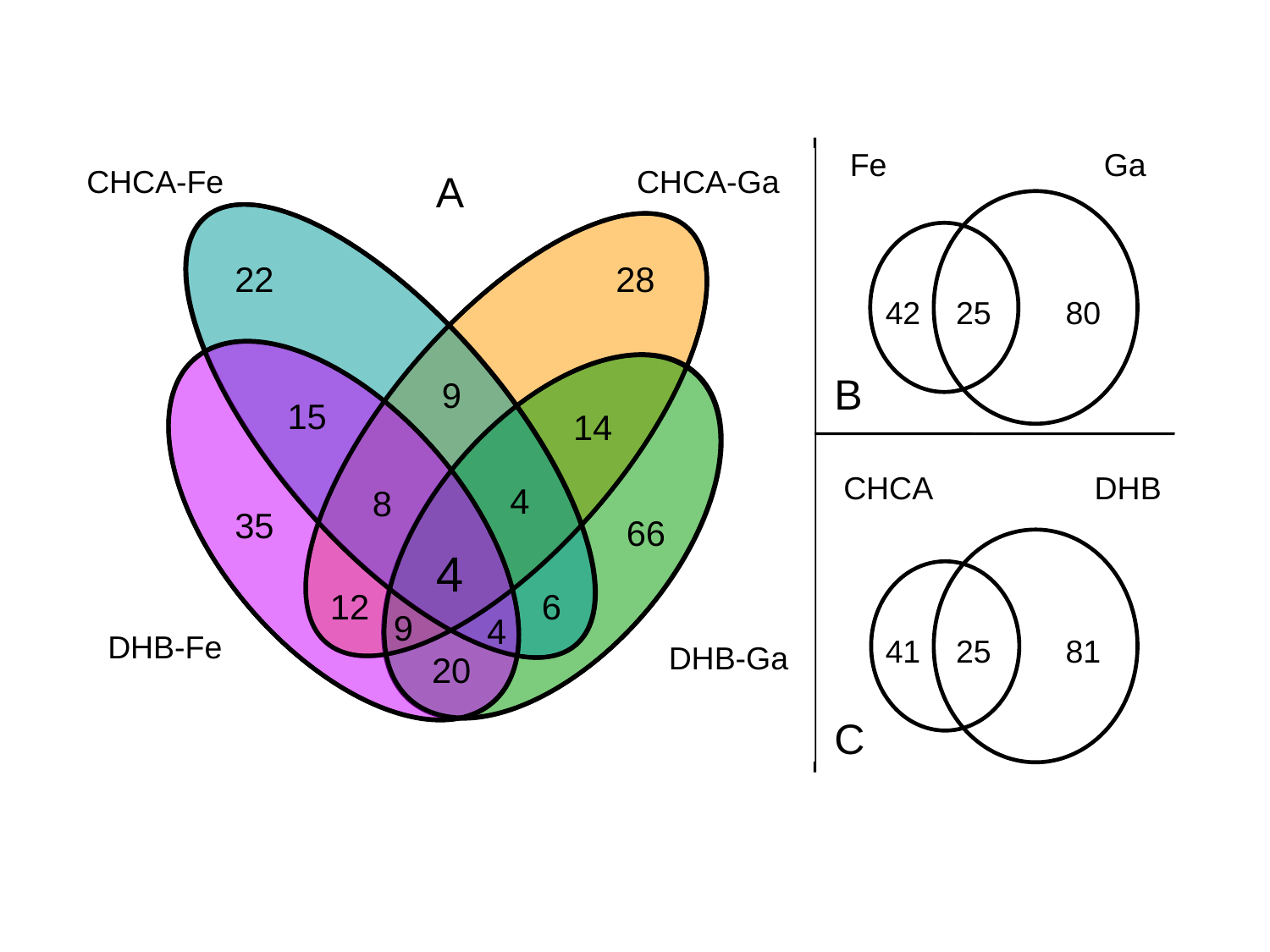

Fe		Ga
CHCA-Fe
CHCA-Ga
A
22
28
42 25	 80
B
9
15
14
CHCA	 DHB
4
8
35
66
4
12
6
9
4
DHB-Fe
41 25	 81
DHB-Ga
DHB-Ga
20
C

Supplement: Figure S1 — Venn diagrams showing m/z peaks overlapping between final m/z peak lists from: (A) four different Mx-Mt combinations, (B) IMAC resins, and (C) MALDI matrices. (0.04 MB PPT) [file pone.0007731.s001.ppt]
