# Supplementary material for: MALDI Profiling of Human Lung Cancer Subtypes
Source: PLoS One. 2009 Nov 5;4(11):e7731. doi: 10.1371/journal.pone.0007731 (PMC2767501; doi:10.1371/journal.pone.0007731)
Supplement: Table S1 — Percentages of correctly classified samples, leave-one out cross-validation percentages of correctly classified samples and m/z peaks included in each Mx-Mt combination discriminant model. Peaks in bold are also included in the 9 m/z peaks global discrimination model. (0.03 MB DOC) [file pone.0007731.s002.doc]

| M-Mc | CHCA-Ga | DHB-Ga | CHCA-Fe | DHB-Fe |
| --- | --- | --- | --- | --- |
| % correctly classified | 98.0% | 89.8% | 75.5% | 83.7% |
| % LOOCV correctly classified | 93.9% | 85.7% | 73.5% | 73.5% |
| m/z peaks included in the model | **1108.15**  1151.79  1350.94  **1670.28**  **1751.56**  **1764.76**  **1798.28**  **1954.77** | 1231.53  1530.4  1536.02  **2127.65**  2275.64  2465.63 | 1516.41  2111.01  2398.77  **3306.43** | 1378.54  1954.6  **2530.23**  1905.63 |
