# Supplementary material for: MALDI Profiling of Human Lung Cancer Subtypes
Source: PLoS One. 2009 Nov 5;4(11):e7731. doi: 10.1371/journal.pone.0007731 (PMC2767501; doi:10.1371/journal.pone.0007731)
Supplement: Table S2 — Differentially expressed peptide masses from the CHCA-MALDI spectra identified by MALDI-TOF/TOF and MASCOT search engine. Individual MASCOT ions scores are significant (p<0.05). (0.03 MB DOC) [file pone.0007731.s003.doc]

| **m/z** | **Mascot score** | **Peptide sequence** | **Protein** |
| --- | --- | --- | --- |
| 1515.96 | 68 | IWHHTFYNELR | β -actin |
| 1668.98 | 119 | VLGAFSDGLAHLDNLK | β -globin |
| 1763.79 | 90 | LISWYDNEFGYSNR | GAPDH |
| 1797.87 | 100 | DVDEAYMNKVELESR | Cytokeratin 8 |
| 1954.08 | 102 | VAPEEHPVLLTEAPLNPK | β -actin |
| 2529.32 | 146 | GTFATLSELHCDKLHVDPENFR | β -globin |
